# Etiology of Functional Nervous Diseases1Read at the meeting of the Medical Society of the County of Niagara held at North Tonawanda, May 1, 1906.

**Published:** 1906-09

**Authors:** James Wright Putnam

**Affiliations:** Buffalo, N. Y.; Professor of Diseases of the Nervous System in the University of Buffalo; Fellow and Ex-president of the American Neurological Association


### BUFFALO MEDICAL JOURNAL.

VOL. LXII. SEPTEMBER, 1906. No. 2

### ORIGINAL COMMUNICATIONS.

### Etiology of Functional Nervous Diseases[1]

BY JAMES WRIGHT PUTNAM, M. D., Buffalo, N. Y.

Professor of Diseases of the Nervous System in the University of Buffalo; Fellow and Ex-president of the American Neurological Association.

THE subject of etiology in functional nervous diseases was chosen because in the neuroses we are sure to find either our greatest successes or our greatest failures. In these cases it is not sufficient to make a diagnosis of the disease but it is equally necessary that we should discover all the etiological factors in a given case in order to successfully treat it. I speak of factors, because in my experience, it is not the rule to find a single cause. The habits and occupation of the patient; the condition of the eyes as regards refractive errors or balance of ocular muscles; the condition of the respiratory gastrointestinal and the genito-urinary tracts; the pelvic organs; the condition of the blood and the urine; mental strain; physical shock; fright ; any of these may act either alone or in varying combinations to produce various clinical pictures of nervous disorders. Each of these causes may produce many symptoms widely dissimilar. We are also liable to find in a series of patients similar symptoms produced by different causes. A study of my cases shows that the various functional diseases with very different symptoms may result from a single cause; that is, one cause may produce different diseases and the reverse is true that several different causes may produce identical symptoms in different patients.

I have found that eye difficulties of various kinds will cause in one patient symptoms of a mental type, in another patient, motor or muscular symptoms will be produced, while in another the symptoms may be purely sensory in character. In a fourth we may have general constitutional disturbances. The mental symptoms will range from simple fatigue after slight mental effort, as reading or writing, to a more serious condition of confusion of thought, forgetfulness, mental irritability, inability to

1. Read at the meeting of the Medical Society of the County of Niagara held at North Tonawanda, May 1, 1906.

concentrate the mind on any subject. Motor symptoms amounting to serious disease, such as chorea, habit spasm, torticollis, have all been observed many times by different clinicians as a result of eye defects.

As to the production of epilepsy by errors of refraction I am still to be numbered among the skeptical. I am mindful of the good results claimed by Dr. Stevens and the late Dr. Ranney in a series of cases of epilepsy treated by refraction, but on the other hand I bear in mind the fact that similar results have not been reported by other observers, and further, that the claims of these gentlemen were fully investigated by a committee of the New York Neurological Society and a report made that those claims of cure were not justified. More recently, at Sonyea Colony for Epileptics in New York no case of epilepsy has been cured by the treatment of the eyes, by such eminent men as Dr. Gould, of Philadelphia and Dr. Bennett, of Buffalo. In my own experience I have frequently come across cases of epilepsy who had coincident eye defects. Some of these have been cured but I have never succeeded in curing the patient by eye treatment alone. To this experience Dr. Spratling of Sonyea, contributes the following note:

"I desire to add a brief clinical note to the paper I published in *American Medicine,* April 9, 1904, 'The Nonoperative Relief of Eyestrain for the Possible Cure of Epilepsy as Tested in 68 Cases at the Craig Colony.' As a result of this test, so painstakingly made by Dr. Gould and Dr. Bennett, one patient only seemed to show a possible recovery from the use of glasses. I can best state the case by quoting from the article in question:

'One male patient (O. B., No. 3), who had 27 attacks between January and September, 1902, had 4 in September, 4 in October, and 4 in December, 12 in all, after he commenced wearing glasses on September 1, 1902. After January, 1903, he had no further attacks up to the date on which this was written, December 1, 1903, a period of 11 months.

His attacks were all grand mal, and of great severity, and were announced some time in advance by a motor aura in the form of a quick, jerking movement in one hand. After a few minutes, the hand, after being lifted higher with each movement, would be jerked straight above the head each time. As soon as the acme of this warning was reached, a similar movement commenced in the left hand, and went through the same order, the attacks appearing after both hands were simultaneously jerked above the head. The patient lives an active, outdoor life, is robust and strong in every respect, and has had epilepsy since his seventeenth year, his present age being 38 years.

He shows a slight degree of epileptic irritability at times, but nothing in the way of permanent mental enfeeblement. He is, or was, an excellent illustration of a motor epileptic, the type in which motor manifestations are marked to the exclusion of those that involve the psychic side. The arrest in his disease now bids fair to pass into complete and lasting recovery.'

It is a matter for regret that the arrest in this patient's disease, which seemed at one time to bid fair to pass into recovery, did not fulfill such a promise. After being fitted with glasses on September 1, 1902, this man had no seizures during the remainder of that or during the following year.

In January, 1904, his glasses were broken, and while not wearing them he had several attacks, 4 in January, 6 in March, and 4 in April. The 6 in March and the 4 in April occurred *after* he had again put on glasses. After April he had no more attacks during 1904, but they recommenced in January, 1905. In that month he had 1, in February, 5."

SENSORY SYMPTOMS CAUSED BY EYE STRAIN.

Headaches are of the first importance. These may be either periodical or continuous, varying or unvarying in severity; occipital, vertical, temporal or frontal in location. They may be lateral or bilateral. They can be angiospastic or angioparalytic in type. All of these different forms of headache may result from different eye difficulties. This has been brought out most prominently by Dr. Gould, in his various articles on headache and eye strain, and notably in his series of biographic clinics. Again, we have as a result of eye strain, general constitutional disturbances of digestion or we may have a symptom grouping of neurasthenia and hysteria. Of this I am certain as a result, not only of my own observation, but of the observation of others, and I have sent patients who have been unable to regain health by other means of treatment, who have made speedy recoveries when the etiological factors of uncorrected eye difficulties had been removed. If, then, uncorrected ocular difficulties will cause symptoms so widely different, surely the correct diagnosing of a disease is not the all important thing. Have we done our duty by diagnosticating neurasthenia or hysteria or migraine or epilepsy or chorea? Must we not go further and seek the cause before we are ready to undertake the cure? Of course all physicians answer "yes, the cause must be sought for and removed." Theoretically, all agree but practically we often see only the empirical method of treatment.

For epilepsy, bromides are prescribed; for chorea, arsenic; for migraine, cannabis indica and migraine tablets. Such prescribing is indeed fruitless if there is in any of these cases a causative factor which has not been discovered, and which if discovered and removed must be the basis of successful treatment. To achieve this in ocular defects, the general practitioner labors at a great disadvantage when he attempts their detection and correction, because when the suggestion is made that the eyes may bear some relation to his disease, we are frequently met by the patient's statement that an oculist has already examined him, that his eyes have been fitted to glasses and that as the symptoms continued the trouble could not be with the eyes. Illustrating this point I refer to a case of a peculiar type of headache that was referred to me from Warren, Pa.

The patient, a man in business, complained of pain over both temporal regions. The pain came on promptly at 9 A. M. and remained until 5 P. M., when it disappeared. This was a daily occurrence, of unvarying severity, and had persisted for a year. After a careful examination I was unable to detect any cause for his headache, but I was impressed by the fact that the headache began when he went to his office and disappeared when his work was done. I decided that the most important cause was an undiscovered eye defect. When I told him this he explained the vision had already been tested and glasses had been put on without benefit. Being still unsatisfied I insisted that glasses were often a misfit, and that oculists varied in their prescriptions for glasses as much as general practitioners would vary in their prescriptions for medicine. I referred him to Dr. Pohlman for examination and prescription. The result was the discovery by Dr. Pohlman of an undetected and uncorrected astigmatism, for which he prescribed the proper lenses. A letter from the patient received some months ago conveyed the glad tidings that he was entirely free from his old headache.

I report this case not because it is unusual, but because it illustrates the fact that the general practitioner is only too frequently at the mercy of the oculist. Many a time in the past few years I have been convinced that certain symptoms were due to a cause connected with the eye in patients who have refused to consult another oculist as they had too frequently been disappointed. In such cases as that I make it a rule first to test the eyes without glasses, and then to test them for reading with the glasses they are at that time wearing, first testing one eye and then the other. In this way it is possible to determine whether the correction gives equal visual reading distance to the two eyes. Frequently the patient will observe that the visual distance for the two eyes differs even with the glasses. If he does notice this it is easier to make him go a second time through the tedious and expensive ordeal of the oculist's office. If my crude examination reveals nothing I persuade or insist, according to circumstances, until he consents to visit an oculist whose work I know and in whom I have confidence.

A case of chorea occurred for three successive Octobers. The case was treated with arsenic and iron, each time the child being taken from school. Then, believing that there might be a causal relation between the chorea and the school work, I sent her to Dr. Grove who found a large refractive error which he corrected. The following October when the patient went to school after her summer vacation she had no attack.

The reported cases of many other physicians will point this same lesson. I am by no means a hobbyist in this matter of eye strain as a causative factor in all functional nervous diseases, but I am a believer in eliminating that as a factor in every case. I have not gone to the length of Dr. Gould, of Philadelphia,, who told me he cured 90% of all his cases of migraine with glasses. His figures exceed mine by a large majority. The reason for this is probably due to the fact that my cases of migraine come to me regardless of the cause. His cases of migraine are sent to him because of a known or suspected ocular defect. I at one time sent him three patients suffering with chronic migraine whom I had been unable to benefit. They were sent to him for the purpose of testing his theory. Not one of these three did he cure.

Another important factor in the causation of functional nervous diseases is chronic constipation and its attendant evil, intestinal fermentation, with its resultant autointoxication. The symptoms which I have seen from this cause have been as varied as can well be imagined. Sleep disturbances are very common. There may be insomnia or sleep disturbed by dreams, while in children we are frequently able to trace night terrors to this cause. Headache, malaise and mental symptoms ranging from irritability and lassitude to melancholia have been observed.

With the intoxication insanities dependant upon a disordered alimentary canal, we have all had some experience, I came near saying some personal experience, for few of us have escaped the mental symptoms known as apathy and disinclination to work, accompanied by some irritability and associated with this same headache. Such symptoms being temporary in character. we have not classified as insanity, but have called them indigestion and constipation, and have cured them by blue pills, salines, abstinence from food and by enforced exercise. But there are cases on record of actual insanities of true intestinal origin. One was by Solders, recorded in the *Jahrbuch für Psychologie,* Bd. xvii.

A woman, when 40 years old, had been constipated for six days, then became suddenly insane, showing a typical picture of confusional mania with excitement. All the symptoms disappeared promptly after the bowel was unloaded.

Berkley records a case of a man 70 years old who, after constipation lasting one week, had an intense headache, this was followed by clouding of mental faculties and the hallucination of a head with a long grey beard beside his own head. Arguments were of no avail. The hallucination persisted until under lavage and saline cathartics he recovered in twenty-four hours and did not relapse.

Epilepsy is largely influenced by the condition of the alimentary canal. So certain am I of this fact that in all my cases of epilepsy I always ask, not only the stock question "Are the bowels regular?" but ask particularly as to the color and odor of the feces.

If these are not normal the patient is put through a course of diet, bowel washes in the knee-chest position and laxatives, until the desired result of a normal stool is attained. A case illustrating this which interested me exceedingly is the following:

In January, 1895, L. B., aged three years was brought to me from Fairport, whose history was as follows: He was one of three children. The others are healthy. In April, 1894, he had a convulsion in which his eyes and head turned to the left. He grew rigid and cold. He had a second one in six weeks; the third in four weeks and in July he had 36 in twenty-four hours. During my first examination he had a stool which to characterise it properly must be called stinking. Its color was very dark, His mother said, "His bowels are regular." My reply was that if they were regular in that way it was no wonder his fits were regular.

He was placed on a careful diet of rice, with bovinine, and malted milk. Salol gr. 3, every 2-4 hours, and ½ teaspoonful of liquid beef peptonoids with creosote were given him six times a day. Aromatic syrup of rhubarb was given at night. As his convulsions were so frequent, ranging from 10 to 20 a day, I thought it best to give him a prolonged rest. He was kept off the floor for six months. His attacks became less frequent, until in July, 1896, they ceased altogether, and his mother writes that he eats, talks and walks as other children do. In this case no bromides were given. In fact I took away the bromides that my predecessor had prescribed.

In all my cases of epilepsy I have carefully watched this relation of bowel condition to severity and frequency of seizures, and have found that in a large majority of cases, attacks increased in frequency when the bowels contained feculent stools, or, in other words, when there was intestinal indigestion.

In direct line with this is a paper by Voisin in the *Archive de Neurologie,* 1895. He reports a number of cases that were treated continuously six months by the bromides alone and then six months by lactose and beta napthol.

He presents the tables in parallel columns of the number of attaques of grand mal and the vertigo under each form of treatment. A study of these tables shows no increase in the number of attacks during the six months in which the bromides were suspended. He presents in all, 18 cases. In some, these attacks were fewer in number, under the napthol than under bromides.

He concludes his study of the cases thus:

We believe that the bromides are especially useful in reflex epilepsy. They diminish the excitability of the patient and place him on guard against his attacks. But in general epilepsy of infectious origin (he here refers to infection from the gastrointestinal tract), we believe that the action of the bromide is insufficient.

Repeated purgatives—intestinal antiseptics, with a good hygiene—life in the country with hydrotherapy appears to us to be the basis of rational treatment.

The urine so often indicates the cause of obscure nervous symptoms that its systematic examination cannot be too strongly insisted upon. It has occurred to me in several cases that a patient suffering from unrelieved headaches had consulted me because my predecessor, the physician in attendance, was satisfied that he had done his entire duty after he had found and corrected an astigmatism. It is a fact that many men, and good men too, are apt to cease looking for causes after they have found one cause. An insufficient secretion of urea is a most common cause of nervous symptoms. These may be mental or motor or sensory in character. The three varieties of these symptoms may be removed by a free use of diuretics and daily liberal doses of distilled water.

Insanity of various types has been observed in both the acute and the chronic varieties of Bright's disease. Insanity is much more frequent in the cases with contracted kidney than it is with the large white kidney. The insanity may appear when the presence of albumin is only indicated as a trace in the urine. The most commonly observed form is the confusional insanity, with hallucinations and with restlessness. So important a fact is this that Berkley says: "The presence of confusional insanity nearly always indicates a toxemia from some source and it behooves the physician to make frequent and accurate examinations of the urine in all suspicious cases in order to avoid unfortunate mistakes in diagnosis." We may also have simple melancholia, or a more active melancholia with distinct delusions of persecution, or we may meet with mania and dementia.

Neurasthenia of a melancholic type is often explained by a urine scanty in amount, of high specific gravity, strongly acid and loaded with urates,—in short, the urine of lithemia. A common cause of this condition is an improper diet and a habit of drinking an insufficient amount of water. This peculiar absence of thirst is quite common in neurasthenics. So marked is this that it is frequently harder to get a patient to drink three pints of water daily than to make them take the most nauseous drugs.

*Anemia* is a frequent cause of headaches, neurasthenia, hysteria and vertigo. It is also so easily discovered that it would be unpardonable in me to more than refer to it before this society, were it not for the fact that it is occasionally overlooked. I will report an extremely interesting case in which anemia,—the cause of all the symptoms was ignored by several able men who had preceded me in the case.

A child was brought to me on account of his having extremely wakeful nights and cross days. He was not only wakeful but noisy. This condition had lasted for two years. The parents were almost worn out. He usually slept until 11, and then would waken very many times till 3 or 4, after which time he remained awake. The physician first consulted was the regular attendant, an able, careful man. He said there must be a reflex irritation and so he had the child circumcised by Dr. Park. This proved of no avail, so Dr. Hinkel was called to examine the nose and throat. These were found in good condition.. The penis was again attacked and this time the meatus urinarius was slit but the child still cried on. The gastrointestinal tract was next viewed with suspicion. The stools were examined but the movements were found digested and normal. The food was regulated but all to no purpose. At last the diagnosis was made of a nervous child with excitable cerebral centers, and bromide of potassium was given faithfully at night, for a long period. This however, was unsatisfactory to parents and physicians as the cause was still undiscovered, and finally the case was referred to me. At first glance I thought I saw a well nourished child. The skin was tanned and gave the face an appearance of ruddy health. On taking hold of the child's arms and legs I was struck with the fact that they were cold. A further look at the mucous membranes of the mouth and eyes showed pallor. The brown cheeks from life in the open air had misled six physicians. It was a case of anemia. The symptoms were all cerebral. The blood examination showed the hemoglobin to be 57 instead of 90. He was placed upon iron three times a day and bovinine added to his food. The bromide which his physician had given him to allay the cerebral irritation was discontinued. In a month the blood examination showed hemoglobin 67—in two months 77. The irritability and wakefulness disappeared.

The case is thus reported in detail, not because it is a new truth but because it is an old one that needs emphasis; that when ill behaved nervous systems are caused by a lack of proper nutrition, the irritation will not down with sedatives, but will continue till the need of the missing element is supplied. The nervous irritability and restlessness is simply the cerebral method of expressing what the nerves express by pain, which Hilton said was the cry of a starved nerve for better food.

*The Causal Relation of the Pelvic Diseases of Women* to the functional nervous diseases must be approached with caution. We must remember that many nervous cases are nervous after the pelvic conditions were completely righted; that women have been told time and again by physicians that if only a lacerated cervix or perineum were repaired or an Alexander's operation performed that the headaches and backaches, nervousness and neurasthenic symptoms would undoubtedly disappear. The operations have been performed with beautiful results, locally, but alas! very often without beneficial results so far as the nervous symptoms are concerned. It has been my experience in the last few years to have a good many women come to me after pelvic operations had been performed, suffering from the same symptoms for which they had undergone operations, in the hope of obtaining relief, proving in their cases there was little or no causal relation between the pelvic condition and the condition of the nervous system. In many cases I have found that there was an element of worry over the fact that something is wrong with the pelvis, and if that worry has been encouraged I believe that it is frequently necessary to invoke gynecological aid.

The safe rule, as it appears to me, is that if there are no local symptoms of irritation it is extremely unlikely that without a local irritation we will have a reflex disturbance. From the fact that in the last 10 years a good many patients have come to me after successful operations by eminent gynecologists, at periods varying from a few months to two years after operations for lacerated cervix or Alexander's operation, with the nervous disturbances unimproved, I have come to the conclusion that the operations, if made, should be made for the local condition only. Then we may save the patient from the blow of bitter disappointment and crushed hopes, as this latter is a mental condition which makes the patient much more difficult for the next man to treat. I do not wish to be misunderstood. I am not arguing against the correction of pelvic disease, or lesions, for that would be folly, but I urge that such pelvic disease should be corrected for its own sake and not make the headache or the insomnia or the irritability of the nervous system the operator's excuse.

As to the relation between pelvic disease and epilepsy, something needs to be said. In my own experience I have not met a case where epilepsy was caused by disease of the uterus or its appendages. I have seen cases in which the attacks occurred at the menstrual period only, and cases that had them more frequently at that time than at any other. But I have not seen cases cured by gynecological interference. In fact, although the statement is often made that epilepsy is frequently caused by disease of ovary or uterus, yet the tables which show it are absent. Neverthless, as epilepsy is such a dreadful and hopeless disease, I am loth to yield a single point, and I endeavor to relieve the attacks with the aid of the gynecologist whenever the opportunity affords.

*Hysteria and Hysterical Convulsions* are, undoubtedly, in many cases the result of intrapelvic disease, and I have occasionally seen cases cured by a suitable operation. It has twice been the operation for the removal of a diseased or prolapsed ovary. While speaking of the causal factor of pelvic disease in nervous diseases of women, it is perhaps appropriate to refer to the fact that in little boys, phymosis is very frequently a cause of obscure functional nervous disease. This has been recognised as far back as 1870, when Dr. Sayre wrote a series of famous papers upon reflex paralysis due to phymosis. Sayre's cases occurred in boys of from three to fifteen years. The symptoms were paresis of the lower limbs of years' duration; constant and painful erections, amounting almost to priapism in some cases, great mental irritability, inability to articulate properly, insomnia, and the like. Relief, usually complete, was afforded by circumcision, the adherent prepuce being torn off from the glans, and the hardened secretions being removed from behind the *corona*. Similar relief was afforded in the case of a girl by clipping the clitoris. Dr. Sayre also saw similar symptoms produced by the irritation of other nerves. Further investigation shows that the irritation may exist in any part of the body. Thorowgood, for example, reports the case of a girl aged 10, suffering from paraplegia, who, on the second day after the expulsion of many ascarides by an aloetic purgative, was able to run and walk, and a few days later was perfectly well.

*Mental Strain from Different Causes.* A very potent factor in this relation, I have left until now, as it is the one about which there can be no argument. That worry over business or practice; that prolonged hours of labor; that neglect of vacation, are causes of severe nervous symptoms is within the experience of all. That the symptoms which these causes induce will be largely mental, the records for the insane will show. The mental symptoms from these causes then will range from insanity down to irritability, inability to work continuously, lack of interest, disturbed sleep. Motor symptoms usually are slight, being expressions of brain disturbance. Still we have restlessness, muscle twitching and tremor. Sensory symptoms are headaches and parasthesia. Secretory symptoms are frequently observed in sweating on slight exertion or during sleep. In these cases woe betide the physician who hopes to cure the case with glasses because he finds astigmatism, or by sewing up a cervix because it is lacerated, or dilating it because it is stenosed. He may do all these but the cause must be met, and rest, and rest only must meet the cause of prolonged strain ere we can claim a victory. Perhaps this is especially impressed upon us by the fact that such patients improve so much when they go to a physician away from home. I know my patients from out of town improve, case for case, much faster than do the patients that I treat in their own homes. The case is the same, the drugs and the correction of reflexes is the same, but the element of removal from the duties of the day is more difficult to enforce at home than abroad.

Fright is more potent as a cause at puberty and more with girls than with boys. This is natural, as it is an emotional cause acting on an emotional sex, at the most emotional age. The cause cannot be removed, is a true criticism. But the cause teaches us a lesson. The nerve centres are easily disturbed. Self poise must be taught. Self control must be practised. This can best be done by systematic work, such as is laid down by rules for feeding at certain hours, walks and massage and baths. Accustom the body to obey rules and subject it to mental control, is the treatment of the nervous patient with an emotional nature. We have so many nervous patients among children that the causes are a most interesting study. Aside from the ones already mentioned, I must add poor home management, for the home life of children has a great influence upon their nerve stability. Children who are petted, whose every whim is gratified, and who learn that their parents cannot resist their tears, lose the benefit of discipline which develops and strengthens character, grow up selfish and self-willed, and when the sorrows and trials come which even indulgent parents cannot remove, they may come to a condition of hysteria.

Children who live in homes where they are constantly abused and distrusted, live, many of them, in a chronic state of dread and apprehension which is so closely allied to hysteria that with an exciting cause it is readily developed. Another and potent cause is the improper diet of children. Among the poor it is customary for the parents to give their little ones tea once or twice a day from the time they leave the breast. So common is this that in my dispensary practice it is a matter of routine with me to ask the mothers whose children suffer with chorea, disorders of sleep, habit spasms and hysterical symptoms, "When did you begin giving the child tea or coffee, and how much does it take?" The majority answer that they commenced to feed them tea and coffee when they were two years old Others have them in their first year. They drink tea two or three times a day. It has seemed to me, that at the time when the intellectual centres are naturally highly stimulated by the new impressions they are daily receiving through each special sense, when they are acquiring the art of language, when memory is being trained to associate names with persons and things, that it is impossible at this period of cerebral growth and development for children to take these cerebral stimulants without their being potent factors in the development of nervous functional diseases.

Per contra, it is said that many are not harmed by this dietary. I do not think this is proven, for we know that a large part of the poorer population is more or less dependent upon stimulants, and it is impossible to tell what influence their early tea drinking may have had in those cases in which a tendency to neuropathic disease exists, because the early giving of these drinks increases the probability of its development. Other causes of hysteria in children may be fright or other profound emotion. Often it comes as a sequel to severe and exhausting diseases and after trauma.

The causes of nervous disease which we call functional are however too numerous to be separately considered. Those that have been chosen have been taken from the long list of etiological factors, because they are representative and because they illustrate the fact that often obscure cases may be exceedingly simple, if a systematic examination be the rule. Often the man with systematic and careful observation will, by his patience, discover causes which perhaps a more scientific and learned man will overlook. But the able man is the one who recognises the cause. Diagnosis is easy. Even the layman will tell you the name of the disease. He will say—headaches, fits, sleeplessness, neuralgia. But it requires the student trained in careful observation and systematic routine of examination to find the cause and so point out the road to recovery.

525 Delaware Avenue.

